# Doses of Immunogen Contribute to Specificity Spectrums of Antibodies against Aflatoxin

**DOI:** 10.3390/toxins9050172

**Published:** 2017-05-19

**Authors:** Peiwu Li, Jing Wu, Li Zhang, Zhiyong Fan, Tingting Yu, Feng Jiang, Xiaoqian Tang, Zhaowei Zhang, Wen Zhang, Qi Zhang

**Affiliations:** 1 Oil Crops Research Institute of the Chinese Academy of Agricultural Sciences, Wuhan 430062, China; peiwuli@oilcrops.cn (P.L.); wujing19900103@sina.com (J.W.); wtxqtutu@163.com (X.T.); zwzhang@whu.edu.cn (Z.Z.); zhangwen@oilcrops.cn (W.Z.); 2 Key Laboratory of Biology and Genetic Improvement of Oil Crops, Ministry of Agriculture, Wuhan 430062, China; 3 Key Laboratory of Detection for Mycotoxins, Ministry of Agriculture, Wuhan 430062, China; 4 Laboratory of Risk Assessment for Oilseeds Products (Wuhan), Ministry of Agriculture, Wuhan 430062, China; 5 College of Food Science and Technology, South China Agricultural University, Guangzhou 510000, China; 6 Hubei Provincial Institute for Food Supervision, No. 666, Gaoxin Road, F District, Wuhan East Lake High-Tech Development Zone, Wuhan 430000, China; siyi-541@163.com (L.Z.); hbzj-fanzhiyong@163.com (Z.F.); cugytt@163.com (T.Y.); yuhuo08@sina.com (F.J.)

**Keywords:** specificity spectrums, dose of immuogen, antibodies, aflatoxin

## Abstract

Research about antibody specificity spectra was conducted to develop single-specific antibodies or broad-specific antibodies. Aflatoxins, as one class of high-toxicity mycotoxins, were selected as the research targets to investigate the effect of the immunogen dose on antibody specificity spectra. For this aim, 16 monoclonal antibodies were induced by low or high doses of aflatoxin B_1_-BSA, and 34 monoclonal antibodies were induced by low or high doses of aflatoxin M_1_-BSA. The specificities of the antibodies induced, whether by aflatoxin B_1_ conjugate or aflatoxin M_1_ conjugate, indicated that the low dose of the immunogen induced a narrow spectrum of antibody specificity, while the high dose of the immunogen showed an advantage to form a broad spectrum of antibody specificity. Therefore, this report provides important information for the development of new antibodies against small molecules like aflatoxins.

## 1. Introduction

Due to immunochemical specificity, using the same immunogen could induce different spectra of antibody specificity. Narrow spectra of antibody specificity are assemblages of specific antibodies that recognize a single target with high specificity. However, broad spectra of antibody specificity are collectives of specific antibodies that can detect various related compounds in one simple test [1,2]. With respect to the research, the specificity spectra of antibodies contributes to the development of narrow-spectrum antibody specificity or broad-spectrum antibody specificity more easily.

Until 2000, researchers put their main efforts into the development of narrow-spectrum antibody specificity [3,4,5]. However, with the advent of congener toxins in food, such as sulfonamides, triazine herbicides, organophosphorus (OP), aflatoxins, etc., multi-analyte determination has attracted considerable interest when screening large numbers of food samples [6,7,8]. Instrumental methods have the potential for simultaneous determination of multiple analogues and may be more specific and sensitive than immunoassays. However, they are expensive and need a larger amount of time for sample preparation before analysis, which has inhibited extending the scope of monitoring, particularly in field-screening scenarios [9]. As an alternative, broad-specificity immunoassays are extraordinarily effective for monitoring and detecting samples of multi-analyte residues in food and environmental samples [10,11], and the development of broad-specificity immunoassays demands the preparation of a broad spectrum of antibody specificity to all target analytes. For example, several attempts have been made to develop broad-specificity immunoassays for OP pesticide residues by the production of a broad-spectrum-specific antibody against OP pesticide [12].

The most commonly used method to produce a broad-specificity immunoassay is to produce an antibody having broad-specificity by using a “generic hapten”, which should demonstrate the common characteristics of all target analytes [12]. Due to the lack of understanding of the specific interactions between antibodies and target analytes or haptens, the antibody specificity resulting from the newly-designed hapten is often unpredictable, and this result comes only after laborious and time-consuming animal experiments. Sometimes an apparent rationally-designed “generic hapten” is unable to generate antibodies with the desirable sensitivity and specificity [13,14,15]. The diverse exposure of an antigenic determinant could form a broad spectrum of the antibody specificity. The most common method of raising the diverse exposure of the antigenic determinant is improve the coupling ratio of the coupling reaction between the hapten and the carrier protein. Another approach to raise the diverse exposure of the antigenic determinant is to use the flexible connection arm with the proper length, and increase the chance of diversity exposure of the hapten. However, the specificity of the antibody was not only impressed by the different structures of the antigen, but also by the immunogenicity or efficiency of the antigen [16], such as the dose of the immunogen. The aim of the present study was to research whether the dose of the immunogen was a key influencing factor to obtain a broad spectrum of antibody specificity or not.

**Figure 1:**
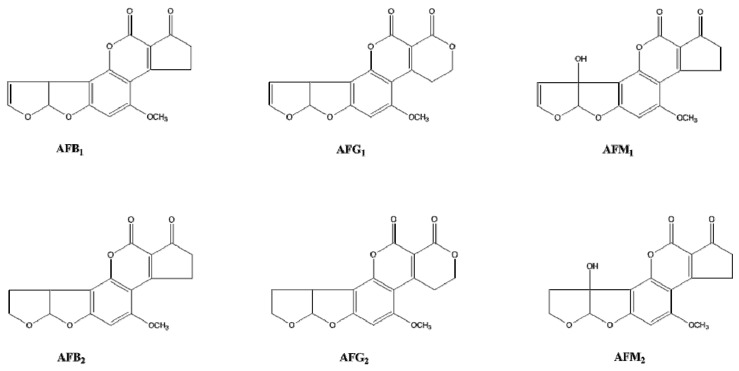
Structures of the main aflatoxins.

Aflatoxins (AF) are members of the coumarin family and have become a main threat worldwide because they are teratogenic, extremely toxic, mutagenic, and carcinogenic. Due to the varying structure of different aflatoxins causing an issue in the development of diagnostic techniques, aflatoxins were chosen as research subjects in this paper [17]. Since all aflatoxins have a similar core structure (Figure 1), it should be possible to develop a single antibody that is able to screening a single target with high specificity, or obtain a generic immunoassay for simultaneous recognition of multiple aflatoxins. Additionally, the strong, rigid structure of aflatoxin molecules are an advantage to study the antigen-antibody interaction [18]. On the other hand, based on 10 years of research on aflatoxins, our team had accumulated a significant amount of hybridoma and monoclonal antibodies, which function against aflatoxins. As it turns out, we found the dose of the immunogen greatly contributes to the specificity spectra of antibodies against aflatoxins. Low doses of the immunogen helped to obtain a narrow spectrum of antibody specificity, while a high dose of the immunogen would help to form a broad spectrum of the antibody specificity.

## 2. Results and Discussion

### 2.1. The Influence on the Dose of the Immunogen against AFB_1_

In each experiment, three female Balb/c mice were subcutaneously immunized with the different doses of the immunogen (AFB_1_-BSA) in multiple sites, and three subsequent subcutaneous injections followed. After the procedure of cell fusion and cloning six times, the stable hybridoma lines were injected into Balb/c hybrid mice and 10–15 mL ascitic fluid was collected from mAbs (monoclonal antibody) from each mouse. Thus, nine mAbs with high sensitivity were obtained in mice immunized with 33 µg every time, and seven were screened successfully in the same way in mice immunized with 150 µg every time.

The mAb specificity was estimated by cross-reactivity (CR) with AFB_2_, AFG_1_, AFG_2_, and AFM_1_ via indirect competitive enzyme-linked immunosorbent assay (ic-ELISA). As the structure of AFB_2_, AFG_1_, AFG_2_, and AFM_1_ are very similar to AFB_1_, they were selected. The value of CR was used to estimate the specificity of ic-ELISA. The cross-reactivity values for different aflatoxins was determined by comparing the IC_50_ values of analytes and calculated according to the following equation: CR (%) = [IC_50_ (AFB_1_)/IC_50_ (analyte)] × 100 [8]. The detailed CR data are summarized in Table 1.

**Figure 2:**
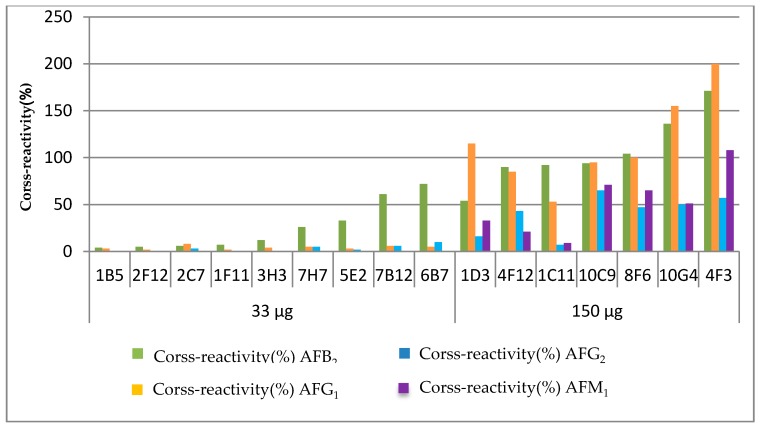
The distribution of specificity spectrums of antibodies against AFB_1_.

The results demonstrate that the CR values of antibodies screened from Mouse Group 1 (the mice immunized with 33 μg) were under 10% when against AFG_1_ and AFG_2_, and showed no cross-reaction with AFM_1_. The cross-reactivity was partly evident against AFB_2_, but the CR values of seven mAbs obtained from Mouse Group 1 were under 50%, only two mAbs were higher than 50%. Compared with Mouse Group 2 (the mice immunized with 150 μg), the dose of the immunogen was nearly five times that of Mouse Group 1, and the specificity spectra of the antibodies were completely different. The seven mAbs that were obtained from Mouse Group 2 showed broad-specificity towards all of the analogues. Figure 2 shows the distribution of the specificity spectra of the 16 antibodies. For example, the CR values of AFB_2_ ranged from 54% up to 171%, and all exceeded 50%. With the low dose of the immunogen, the CR values of AFB_2_ ranged from 4 to 72%. It seems that the mAbs obtained from Mouse Group 1 showed narrow spectra of antibody specificity, and were broader with the higher dose of the immunogen.

### 2.2. The Influence on the Dose of the Immunogen against AFM_1_

To verify whether the dose of the immunogen contributes to the specificity spectra of the antibodies against aflatoxin or not, a similar test was conducted which used AFM_1_ as the analyte. Based on works we had conducted before, the dose of the immunogen, comparing between 16 µg per Balb/c mice and 65 µg per Balb/c mice, resulted in us obtaining 34 kinds of monoclonal antibodies against AFM_1_. The aflatoxins AFB_1_, AFB_2_, AFG_1_, and AFG_2_ as analogues of AFM_1_ were tested for cross-reactivities (CR). The sensitivity and cross-reactivity are displayed in Table 2. 

**Figure 3:**
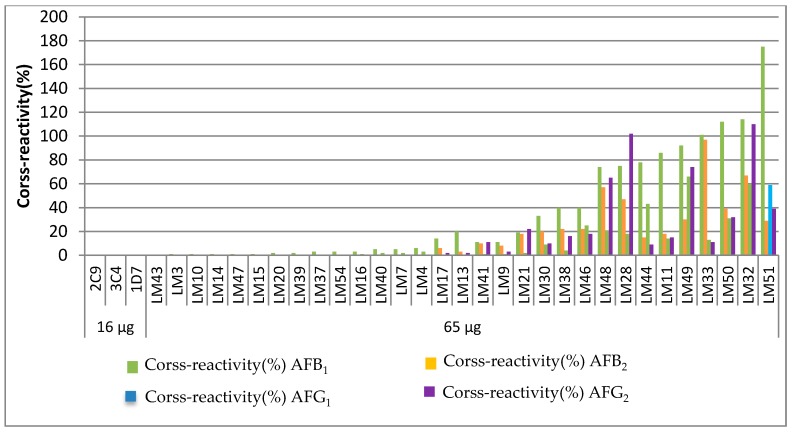
The distribution of specificity spectrums of antibodies against AFM_1_.

The sensitivities of antibodies were basically similar, but the cross-reactivities were different. No significant cross-reactivity was observed against aflatoxin B_1_, B_2_, G_1_, and G_2_ for the mAb of 2C9, 3C4, or 1D7. However, when the dose of the immunogen was increased from 16 µg to 65 µg, the specificity spectra of antibodies changed. The antibodies showed good cross-reactivity and could be classified broadly into five distinct groups. LM43 was assigned to Group 1, and the CR value was equal to that of 2C9 mAb, which could not cross-react with aflatoxin B_1_, B_2_, G_1_, and G_2_. Nine monoclonal antibodies were assigned to Group 2: LM3, LM10, LM14, LM47, LM15, LM20, LM39, LM37, and LM54, which had weak reaction efficiency with B_1_ but showed no cross-reaction with B_2_, G_1_, G_2_. Group 3 contained four monoclonal antibodies, LM16, LM40, LM7, and LM4, which had low CR values of B_1_ and G_1_ and showed no cross-reaction with B_2_ and G_2_. LM17 and LM13, with low CR values of B_1_, B_2_, and G_1_, were assigned to Group 4. The remaining were assigned to Group 5, which had similar reaction intensities with the other four aflatoxins. In particular, the antibody LM32 showed a high reactive specificity and sensitivity for the five toxins, as well as cross-reactivity with closely-related toxins. Longitudinally, when the dose of the immunogen increased to 65 µg, the CR values of AFB_1_ ranged from 1 to 175%, the CR values of AFB_2_ ranged from 1 to 97%, the CR values of AFG_1_ ranged from 1 to 66%, and the CR values of AFG_1_ ranged from 1 to 110%. We can draw a conclusion from the data of the five distinct groups that the higher the dose of the immunogen resulted in a wider range of CR values. Figure 3 shows the distribution of the specificity spectra of five types of antibodies. All of the results are in agreement with what might have been predicted by using different doses of the immunogen that exhibited different specificity spectra of antibodies. The greater the dose of the immunogen, the wider the specificity spectra of the antibodies may be.

### 2.3. Discussion

**Figure 4:**
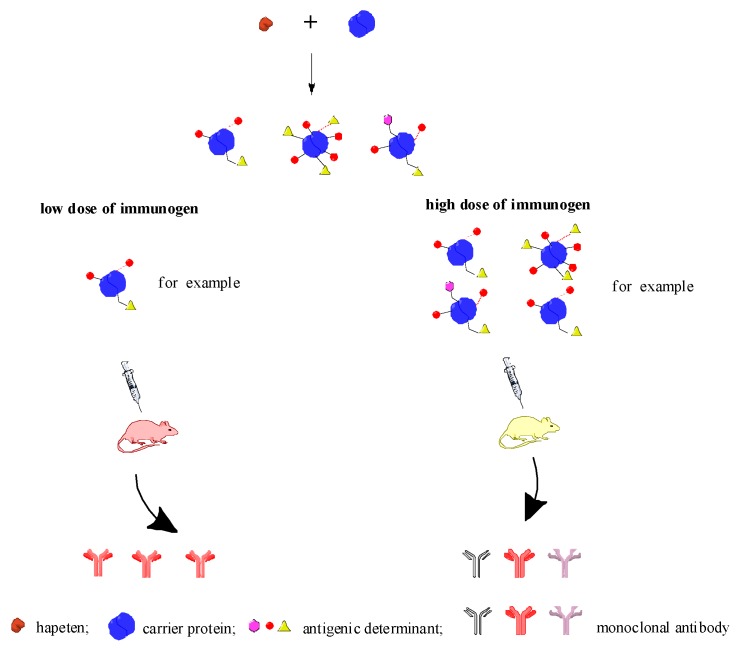
Model for different dose of immunogen acting on Balb/c mice.

The study provides a potential method for the development of narrow/broad spectra of antibody specificity by controlling the dose of the immunogen. Antibody–antigen interactions are indispensable to immunoassay, although the interactions at the molecular level are, in general, undetermined. It is suggested that the antigen–antibody recognition is based on steric criteria and on interactions resulting from the electronic properties of the molecules [19], so the position space or quantity of antigen epitopes may play a significant role in antigen–antibody interaction. For example, in the sketch in Figure 4, the same hapten conjugates to the carrier protein may turn up different antigen epitopes, which means the antigenic determinants are different, with the difference containing the length of the spacer arm, position space of the antigen epitopes, the coupling ratio of the hapten and the carrier protein, etc. When increasing the dose of the immunogen, the antigenic determinant became more diversely exposed. Thus, the opportunity to select various monoclonal antibodies arose, which means a low dose of the immunogen was helpful to obtain a narrow spectrum of antibody specificity, while a high dose of the immunogen helped to form a broad spectrum of antibody specificity.

## 3. Conclusions

In this study, the contribution to the specificity spectra of antibodies against aflatoxins was discussed. It turned out that a low dose of the immunogen helped obtain a narrow spectrum of antibody specificity while a high-dose of the immunogen helped to form a broad spectrum of antibody specificity. To verify the point, we used different doses of the immunogen to develop zearalenone mAb and capsaicine mAb, and the result was found to be consistent.

In conclusion, the findings provide a foundation for the development of specificity spectra of antibodies and for the establishment of broad-spectrum rapid screening methods for toxins.

## 4. Experimental Section

### 4.1. Chemicals and Instruments

Aflatoxin M_1_-BSA conjugate (Lot # 083m4109v, 4.24 mole AFM_1_ per mole BSA, coupling through active ester with engineered Aflatoxin M_1_), aflatoxin B_1_-BSA conjugate (Lo t# 093m405080, 8.7 mole AFB_1_ per mole BSA, coupling through active ester with engineered Aflatoxin B_1_), Aflatoxin M_1_, B_1_, B_2_, G_1_, and G_2_ standard solution, incomplete and complete Freund’s adjuvants, 3,3′,5,5′-tetramethylbenzidine (TMB), goat anti-mouse IgG-horseradish peroxidase (IgG-HRP), polyethylene glycol 1450, hypoxanthine-thymidine, and hypoxanthine–aminopterin–thymidine, were all obtained from Sigma-Aldrich(St. Louis, MO, USA). Fetal bovine serum, streptomycin (10,000 Lg/mL), and penicillin (10,000 U/mL) were from Gibco. RPMI-1640 medium, l-glutamine, and HEPES (2-[4-(2-Hydroxyethyl)-1-piperazinyl]ethanesulfonic acid) were from HyClone. All other reagents were of analytical reagent grade or better, unless otherwise stated. Female Balb/c mice were obtained from the Centers for Disease Control and Prevention of Hubei Province. Water was obtained from a MilliQ purification system.

Cell culture plates were purchased from Iwaki Co. (Iwaki, Japan). Absorbance was measured at a wavelength of 450 nm using a SpectraMax M2e microplate reader from PerkinElmer (Waltham, MA, USA). Polystyrene 96-well microtiter plates were obtained from Costar (Cambridge, MA, USA). The mice were approved by The Laboratory Animal Monitoring Committee of Hubei Province (identification code: 42000600015661; date of approval: 2016.07.05).

### 4.2. Immunization

In the first immunization, different doses of the immunogen were dissolved in sterilized 0.85% NaCl solution and then emulsified with an equal volume of Freund’s complete adjuvant, and the final water-in-oil emulsion was injected into multiple sites subcutaneously into three eight-week-old female Balb/c mice.

Different doses of the immunogen were conducted with the same doses using Freund’s incomplete adjuvant and injected on the fourth week, seventh week, and ninth week after the initial immunization. At the seventh day after the fourth injection, antisera were gathered from the caudal vein of each mouse and assayed for anti-aflatoxin B_1_ or M_1_ antibodies by indirect competitive ELISAs (ic-ELISAs) with aflatoxin M_1_, B_1_, B_2_, G_1_, and G_2_ as the competitors. The mice whose antiserum exhibited higher sensitivity were given an intraperitoneal booster three days before the spleen was removed. The booster injection used a two-fold dose of antigen without emulsification with adjuvant.

### 4.3. Production of mAbs

The hybridoma cells were obtained by fusion of SP2/0 murine myeloma cells with the spleen cells isolated from the selected mice using PEG 2000 [20]. After cell fusion, when the hybridoma cells were grown to approximately 40% confluence in wells at 7–10 days, culture supernatants were collected and screened using indirect ELISA for the presence of antihapten antibodies. Selected hybridomas were cloned by limiting dilution, and stable antibody-producing clones were expanded. An indirect competitive ELISA (ic-ELISA) was employed to screen if the antibodies could recognize the analytes. Antibodies were generated by ascites growth using the selected clones. Ascites fluids were collected and purified using the method of caprylic acid-ammonium sulfate precipitation and were used in the following ELISA [21]. 

### 4.4. Evaluation of Antibody Sensitivity and Cross-Reactivity

The indirect competitive ELISA format as described was used to evaluate the sensitivity of each monoclonal antibody. The procedure of the ic-ELISA was as follows: flat-bottom polystyrene ELISA plates were coated with AFM1-BSA/AFBI-BSA (100 μL/well) in carbonate buffer (pH 9.6) at 37 °C overnight, and the each well was blocked with 200 μL 4% skim milk in PBST solution at 37 °C for 1 h. Each well was incubated with 50 μL of the analyte in methanol-PBS and 50 μL of optimized dilutions of antibodies were added. After incubation at 37 °C for 40 min, goat anti-rabbit IgG-HRP diluted to 1:5000 was added (100 μL/well) to each well, and the plates were incubated for 30 min at 37 °C. Then, 100 μL per well of TMB solution was added and incubated for 10 min at 37 °C. After each step, a PBST washing step was carried out. The reaction was stopped by addition of 50 μL of 2 M H_2_SO_4_ and the OD values were recorded at 450 nm. The optimum dilution of antibody required as a working concentration was defined as the dilution which gave an absorbance closest to 1.0 [22]. Each competition reaction was carried out in duplicate with at least seven concentrations of aflatoxin, and the last well was blank to contrast. %B/B_0_ could express these data while the absorbance in the absence of analyte was B_0_, and the value of B represents the absorbance at each concentration of analyte. The cross-reactivity values, CR, for different aflatoxins was determined by comparing the IC_50_ values of the analytes and calculated according to the following equation: % CR = (IC_50_ AFB_1_ or AFM_1_/IC_50_ analyte) × 100 [23].

## Figures and Tables

**Table 1:** Comparison of the cross-reactivity of anti-AFB_1_ antibodies.

Dose of Immunogen	mAb	IC_50_ for AFB_1_ (ng mL^−1^)	Cross-Reactivity (%)				
(µg per Balb/c Mice)			AFB_1_	AFB_2_	AFG_1_	AFG_2_	AFM_1_
33	1B5	0.012	100	4	3	<0.1	<0.1
33	2F12	0.010	100	5	2	0.2	<0.1
33	2C7	0.020	100	6	8	3	<0.1
33	1F11	0.052	100	7	2	<0.1	<0.1
33	3H3	0.023	100	12	4	<1	<0.1
33	7H7	0.052	100	26	5	5	<0.1
33	5E2	0.013	100	33	3	2	<0.1
33	7B12	0.012	100	61	6	6	<0.1
33	6B7	0.027	100	72	5	10	<0.1
150	1D3	0.44	100	54	115	16	33
150	4F12	0.086	100	90	85	43	21
150	1C11	0.001	100	92	53	7	9
150	10C9	2.09	100	94	95	65	71
150	8F6	1.70	100	104	100	47	65
150	10G4	0.73	100	136	155	50	51
150	4F3	0.29	100	171	200	57	108

**Table 2:** Comparison of the cross-reactivity of anti-AFM_1_.

Dose of Immunogen (µg per Balb/c Mice)	mAb	IC_50_ for AFM_1_ (ng mL^−1^)	Cross-Reactivity (%)				
			AFM_1_	AFB_1_	AFB_2_	AFG_1_	AFG_2_
16	2C9	0.067	100	<1	<1	<1	<1
16	3C4	0.043	100	<1	<1	<1	<1
16	1D7	0.058	100	<1	<1	<1	<1
65	LM43	0.014	100	<1	<1	<1	<1
65	LM3	0.029	100	1	<1	<1	<1
65	LM10	0.034	100	1	<1	<1	<1
65	LM14	0.017	100	1	<1	<1	<1
65	LM47	0.020	100	1	<1	<1	<1
65	LM15	0.006	100	1	<1	<1	<1
65	LM20	0.011	100	2	<1	<1	<1
65	LM39	0.069	100	2	<1	<1	<1
65	LM37	0.017	100	3	<1	<1	<1
65	LM54	0.029	100	3	<1	<1	<1
65	LM16	0.030	100	3	<1	1	<1
65	LM40	0.029	100	5	<1	2	<1
65	LM7	0.052	100	5	<1	2	<1
65	LM4	0.014	100	6	<1	3	<1
65	LM17	0.069	100	14	6	<1	2
65	LM13	0.011	100	20	3	<1	2
65	LM41	0.015	100	11	10	1	11
65	LM9	0.014	100	11	8	1	3
65	LM21	0.011	100	19	18	2	22
65	LM30	0.023	100	33	20	9	10
65	LM38	0.014	100	40	22	4	16
65	LM46	0.011	100	40	22	25	18
65	LM48	0.012	100	74	57	21	65
65	LM28	0.012	100	75	47	18	102
65	LM44	0.019	100	78	15	43	9
65	LM11	0.019	100	86	18	14	15
65	LM49	0.019	100	92	30	66	74
65	LM33	0.013	100	101	97	13	11
65	LM50	0.035	100	112	39	31	32
65	LM32	0.046	100	114	67	60	110
65	LM51	0.023	100	175	29	59	39
